# Association of CSF soluble TREM1 levels with hippocampal atrophy in cognitively impaired older adults

**DOI:** 10.3389/fnagi.2024.1481526

**Published:** 2025-01-14

**Authors:** Hao Shu, Gangyu Ding, Xiaona Xu, Xuerong Huang, Ruqian He

**Affiliations:** ^1^Department of Neurology, The Third Affiliated Hospital of Wenzhou Medical University (Ruian People’s Hospital), Wenzhou, Zhejiang, China; ^2^Department of Neurology, Jiading District Central Hospital Affiliated Shanghai University of Medicine and Health Sciences, Jiading, Shanghai, China

**Keywords:** CSF sTREM1, neuroinflammation, hippocampal atrophy, cognitive impairment, Alzheimer’s disease

## Abstract

**Background:**

Recent studies have shown that cerebrospinal fluid (CSF) levels of soluble triggering receptor expressed on myeloid cells 1 (sTREM1) are elevated in individuals with Alzheimer’s disease (AD), though the relationship between CSF sTREM1 and hippocampal atrophy remains to be elucidated. The primary aim of this study was to investigate the association between CSF sTREM1 levels and longitudinal changes in hippocampal volumes, and to determine if this relationship is moderated by cognitive status.

**Methods:**

We included 576 participants, comprising 152 cognitively unimpaired (CU) and 424 cognitively impaired (CI) individuals. In the cross-sectional analyses, Pearson’s correlation tests were conducted to examine the relationship between baseline CSF sTREM1 levels and hippocampal volumes in both CU and CI participants. For the longitudinal analyses, a linear mixed-effects model was employed to assess the significance of the three-way interaction between CSF sTREM1 levels, cognitive status, and follow-up time on adjusted hippocampal volume (aHV). Further stratified analyses based on cognitive status were performed to dissect the specific effects within each group.

**Results:**

Our findings revealed significantly elevated baseline CSF sTREM1 levels in CI participants compared to CU participants. Cross-sectional analyses demonstrated that CSF sTREM1 levels were negatively associated with hippocampal volumes in both CU and CI participants. In the longitudinal analyses, the three-way interaction between CSF sTREM1 levels, cognitive status, and follow-up time was found to be significant for aHV. Stratified analyses indicated that, in CI participants, higher CSF sTREM1 levels were associated with a more accelerated rate of hippocampal atrophy, whereas no such association was observed in CU participants.

**Conclusion:**

These results underscore the complex interplay between neuroinflammation, as reflected by CSF sTREM1 levels, hippocampal atrophy, and cognitive decline. The data suggest that neuroinflammation may contribute differently to hippocampal atrophy rates in CI versus CU individuals, highlighting the potential for targeted anti-inflammatory interventions in the prevention and treatment of AD.

## 1 Introduction

Alzheimer’s disease (AD) is a neurodegenerative disease that progressively impairs cognitive functions, with memory impairment being a primary symptom (Scheltens et al., 2021). The disease is hallmarked by the accumulation of amyloid-beta (Aβ) plaques, neurofibrillary tangles, and atrophy of critical brain regions (Scheltens et al., 2021). The hippocampus, a key structure for learning and memory (Kilpatrick et al., 1997; Eldridge et al., 2000), is particularly vulnerable to the degenerative processes associated with AD, making its volume a well-established marker for disease progression (Jack et al., 2010; Landau et al., 2010; Kantarci et al., 2013). Early detection and understanding of the mechanisms underlying hippocampal atrophy are crucial for the development of novel interventions and therapies.

The critical role of neuroinflammation in AD has attracted growing interest (Calsolaro and Edison, 2016). Microglia play a critical role in the surveillance and response to neuropathological changes (Cai et al., 2022; Wu and Eisel, 2023). The triggering receptor expressed on myeloid cells 1 (TREM1) is a glycoprotein receptor mainly expressed by microglia and monocytes (Saadipour, 2017). Elevated levels of sTREM1 have been reported in the cerebrospinal fluid (CSF) and plasma of individuals with AD compared to controls (Jiang et al., 2019; Del Campo et al., 2022; Hok et al., 2023), suggestive of a relation between microglial activation and disease progression. Polymorphisms in the *TREM1* gene have been related with the aggregation of neuritic and amyloid plaques and a steeper cognitive decline (Replogle et al., 2015). To our knowledge, however, the relationship between CSF sTREM1 levels and longitudinal hippocampal atrophy remains unknown.

In the current study, we aimed to study the relationship between CSF sTREM1 levels and the rate of hippocampal atrophy over time in older individuals who are either cognitively unimpaired (CU) or cognitively impaired (CI). This research could provide important insights into the role of sTREM1 in the neurobiology of AD and may contribute to the development of new diagnostic and therapeutic strategies. Our hypothesis was that higher CSF sTREM1 levels would be associated with a more rapid rate of hippocampal atrophy in older individuals, particularly in CI individuals. By examining this relationship, the current work may help better understand the interplay between neuroinflammation, brain structure changes, and cognitive decline.

## 2 Methods

### 2.1 Alzheimer’s Disease Neuroimaging Initiative database

Cross-sectional and longitudinal data used in the current study were extracted from the ADNI database.^1^ The ADNI was launched in 2003 as a public-private partnership. The primary aim of ADNI has been to examine whether a combination of methods, such as cognitive assessments, magnetic resonance imaging (MRI) techniques, positron emission tomography (PET) measurements, and other biological markers, can be used to track the clinical progression of mild cognitive impairment (MCI) and early AD. For other detailed information about ADNI, see www.adni-info.org. The ADNI study was approved by the Institutional Review Boards at each participating site, and written informed consent was provided by each study participant or their authorized representatives.

### 2.2 Participants

In the current study, we selected subjects who had at least two MRI measurements of hippocampal volume and had baseline measurements of CSF sTREM1 levels. There were 576 study participants, including 152 CU subjects and 424 CI subjects, comprising MCI and mild AD dementia. Specific enrollment criteria have been described previously (Aisen et al., 2024) and can also be found on the website.^2^ In addition, these criteria have been used in previous publications (Sundermann et al., 2016; Wang et al., 2023). Briefly, the criteria for CU included a Mini-Mental State Examination (MMSE) (Folstein et al., 1975) score of 24 or higher and a Clinical Dementia Rating (CDR) (Morris, 1993) score of 0. The criteria for MCI included an MMSE score of 24 or higher, a CDR score of 0.5, a subjective memory complaint, objective memory impairment as measured by the Wechsler Memory Scale Logical Memory II, and essentially preserved abilities to conduct daily life activities. The criteria for mild AD dementia included an MMSE score between 20 and 26, a CDR score of 0.5 or 1, and meeting the National Institute of Neurological and Communicative Disorders and Stroke-Alzheimer’s Disease and Related Disorders Association criteria for probable AD (McKhann et al., 1984).

### 2.3 Measurement of CSF sTREM1, soluble triggering receptor expressed on myeloid cells 2 (sTREM2), and macrophage migration inhibitory factor (MIF) levels

CSF sTREM1, sTREM2, and MIF levels were determined as a part of proteomic analytes using SomaLogic’s SomaScan platform by the Neurogenomics and Informatics Center at Washington University. The preliminary standardization processes for SOMAscan protein quantifications were carried out by SomaLogic. In essence, the hybridization normalization was executed on a per-sample basis. The aptamers were subsequently categorized into three distinct normalization cohorts—designated S1, S2, and S3—this categorization was informed by the signal-to-noise ratio observed in both technical replicates and samples. This stratification was critical to prevent the amalgamation of aptamers with disparate protein signal intensities during subsequent normalization phases. Following this, a median-based normalization was applied to mitigate various assay-related inconsistencies, including variations in protein concentration, pipetting, reagent concentration, and timing of the assay (Candia et al., 2017). Levels of CSF markers are expressed in relative fluorescence unit (RFU).

### 2.4 Measurement of CSF AD biomarkers

Lumbar puncture and sample collection were performed as detailed in the ADNI manual.^3^ Levels of CSF AD biomarkers, including CSF Aβ42 and phosphorylated-tau at threonine 181 (p-tau181), were examined by the Roche Elecsys Aβ42 CSF and Elecsys p-tau CSF immunoassays at the Department of Pathology & Laboratory Medicine and Center for neurodegenerative Diseases Research, Perelman School of Medicine University of Pennsylvania (UPENN). Details of the methods and procedures have been described elsewhere (Bittner et al., 2016). Levels of CSF Aβ42 and p-tau181 were expressed as pg./ml.

### 2.5 Determination of APOE4 genotype

APOE (gene map locus 19q13.2) genotypes of the study participants were extracted from the ADNI database. Detailed information on blood sample collection and genotyping processes can be found on the ADNI website (see text footnote 1). Participants with at least one ɛ4 allele were classified as APOE4 carriers, and those with no ɛ4 allele were categorized as APOE4 non-carriers.

### 2.6 Hippocampal volumetric MRI measures

The methodology pertaining to the acquisition of MRI data has been detailed previously (Jack et al., 2008). Utilizing either a 1.5 T or 3 T scanner, scans were conducted following a uniform protocol that underwent validation across different locations. The imaging protocol included the collection of high-resolution, T1-weighted images using a volumetric magnetization-prepared rapid gradient echo sequence in the sagittal plane, as well as T2-weighted images using a fast-spin echo sequence in the axial plane. Before initiating data collection, customized imaging protocols were specifically developed and confirmed for accuracy through testing on both phantom models and in 137 human subjects. For each participant, a phantom scan was also performed to ensure an optimal signal-to-noise ratio, with centralized assessment for quality control. Further details on the validation procedures can be found on the ADNI website.^4^ Adjustments for sex-related variations in head size were made by computing the adjusted hippocampal volume (aHV), determined through the calculation: [(hippocampal volume/intracranial volume) * 1000]. As a result, the aHV indicates the proportional extent of gray matter volume in the regional context.

### 2.7 Statistics

Baseline demographic characteristics and variables of interest (CSF sTREM1 levels and aHV) were compared between disease stages (CU vs. CI) using Welch’s two-sample *t*-tests for continuous variables and Pearson’s chi-squared tests for categorical variables. Pearson’s correlation tests were employed to assess the relationship between baseline CSF sTREM1 levels and aHV separately in CU and CI individuals. To explore whether baseline CSF sTREM1 levels influence hippocampal atrophy longitudinally and whether this effect varies by disease stage, several linear mixed-effects models were applied. Models were performed separately for CU and CI participants. The model specified repeatedly measured aHV as the outcome variable and the interaction term among continuous CSF sTREM1, disease stage (CU vs. CI), and follow-up time (years) as the focal predictor. Additional covariates included in the model were baseline age, gender, education, APOE4 carrier status (non-carriers vs. carriers), amyloid status [Aβ-negative vs. Aβ-positive; participants with CSF Aβ42 levels <1,098 pg./mL were classified as Aβ-positive based on previous literature (Schindler et al., 2018)], and their interactions with follow-up time. Models included the main effects of predictors and their interactions with time. The model incorporated a random intercept to account for individual variability. The linear mixed-effects model is summarized using the following equation:


$$\begin{array}{l} {aHV}_{\mathrm{change}}\;∼CSF\;\mathrm{sTREM1}*\mathrm{time}+\mathrm{Age}*\mathrm{time}+\mathrm{Gender}*\mathrm{time}\\ +\mathrm{Education}*\mathrm{time}+\mathrm{APOE4 status}*\mathrm{time}\\ +\mathrm{Amyloid status}*\mathrm{time} \end{array}$$

where aHV_change_ is the change in aHV from the baseline.

As a sensitivity analysis, we included raw hippocampal volumes, rather than adjusted volumes, as outcomes in the linear mixed-effect models. We conducted two separate linear mixed-effect models for the CU and CI groups. All statistical analyses were performed using R software (The R Core Team, 2014).

## 3 Results

### 3.1 Sample characteristics by cognitive status

At baseline, the study comprised 576 participants, consisting of 152 CU and 424 CI individuals. Significant differences were observed between the CU and CI groups for most demographic and clinical variables, with the exception of years of education and the percentage of female participants (see Table 1). For instance, CI participants exhibited higher levels of CSF sTREM1 and reduced hippocampal volumes relative to CU participants. As anticipated, the CI group had a greater frequency of APOE4 carriers, and a higher prevalence of amyloid positivity compared to the CU group. Additionally, the average follow-up duration was shorter for CI participants than for those who were cognitively unimpaired. To alleviate the potential impact of sample imbalances, we randomly selected 152 out of the total 424 CI participants and then compared CSF sTREM1 levels between the CU and CI groups using two-sample *t*-tests. The results remained unchanged: the mean sTREM1 level was 216.04 in the CU group and 232.5 in the CI group (*t* = −4.28, df = 286.16, *p* < 0.001).

**Table 1:** Sample characteristics by cognitive status.

Characteristic	Overall, *N* = 576	CU, *N* = 152	CI, *N* = 424	*p*-value
Age, years	73 (7)	74 (6)	73 (8)	0.002
Education, years	16 (3)	16 (3)	16 (3)	0.10
Gender				0.3
Male	326 (57%)	81 (53%)	245 (58%)	
Female	250 (43%)	71 (47%)	179 (42%)	
APOE4 status				<0.001
APOE4 noncarriers	302 (52%)	118 (78%)	184 (43%)	
APOE4 carriers	274 (48%)	34 (22%)	240 (57%)	
MMSE score	27 (3)	29 (1)	27 (3)	<0.001
Follow-up duration, years	3.24 (2.77)	4.76 (3.27)	2.94 (2.39)	< 0.001
aHV	4.50 (0.80)	4.95 (0.54)	4.34 (0.82)	<0.001
Amyloid status^a^				<0.001
Amyloid negative	207 (36%)	96 (63%)	111 (26%)	
Amyloid positive	369 (64%)	56 (37%)	313 (74%)	
Amyloid status^b^				<0.001
Amyloid negative	241 (42%)	106 (70%)	135 (32%)	
Amyloid positive	335 (58%)	46 (30%)	289 (68%)	
sTREM1, RFU	227 (35)	216 (29)	231 (37)	<0.001

Continuous variables are summarized as Mean (SD), while categorical variables are summarized as n (%). Comparisons between two groups for continuous variables were conducted using Welch’s Two Sample *t*-tests, and comparisons for categorical variables were performed using Pearson’s Chi-squared tests. CU, cognitively unimpaired; CI, cognitively impaired; APOE, apolipoprotein E; MMSE, Mini-Mental State Examination; aHV, adjusted hippocampal volume; sTREM1, soluble Triggering Receptor Expressed on Myeloid Cells 1; RFU, Relative Fluorescence Unit.

a CSF Aβ42 levels < 1,098 pg/mL was used to classify amyloid positivity.

b CSF p-tau181/Aβ42 ratio > 0.0198 was used to classify amyloid positivity according to a previous study (Schindler et al., 2018).

### 3.2 Relationship between baseline CSF sTREM1 levels and hippocampal volume in the CU and CI groups

**Figure 1:**
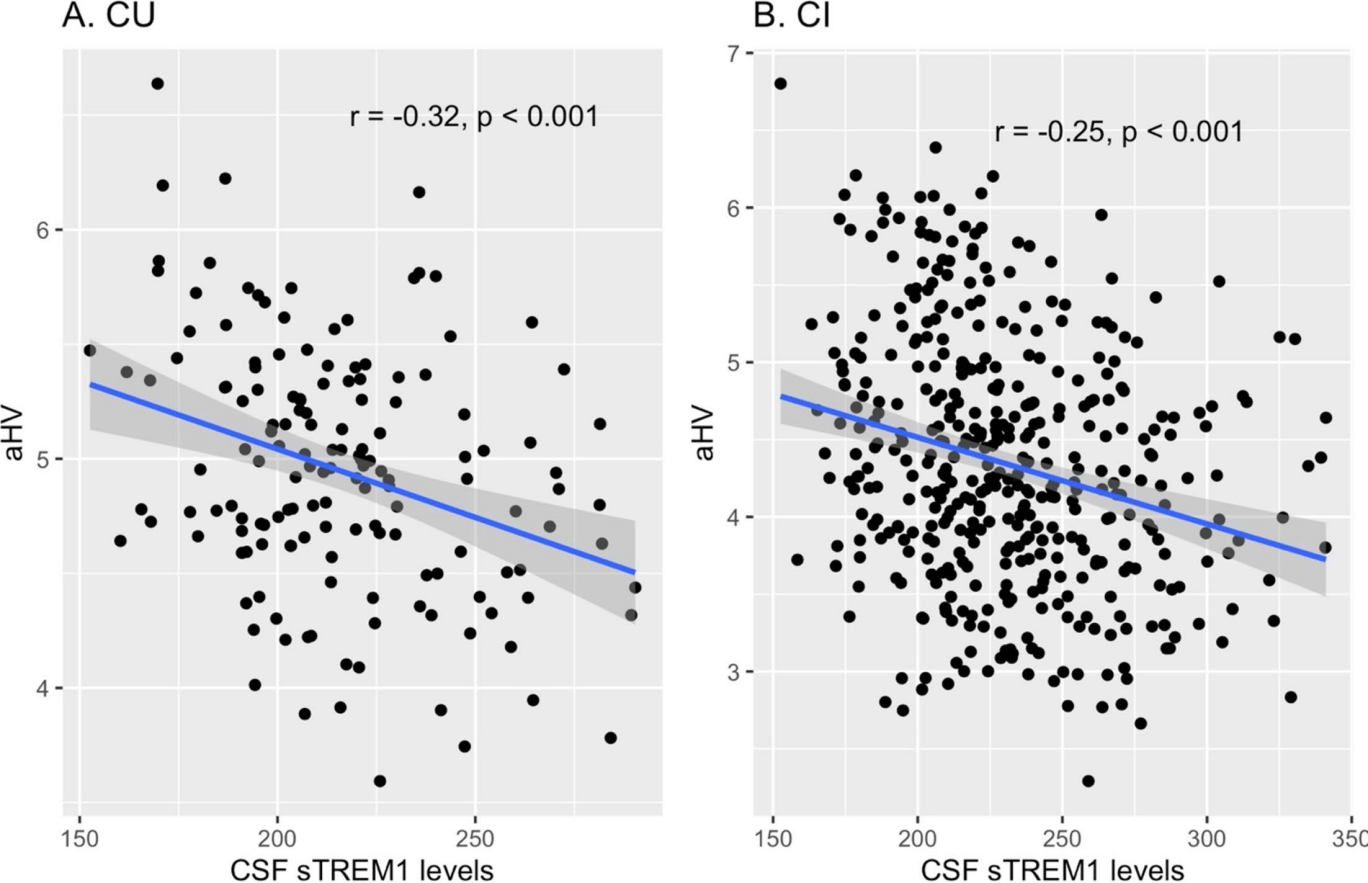
Cross-sectional relationships between baseline CSF sTREM1 levels and hippocampal volumes in the CU and CI groups. **(A)** Illustrates the relationship between CSF sTREM1 levels and hippocampal volumes in CU participants (*r* = −0.32, *p* < 0.001), while **(B)** shows this relationship in CI participants (*r* = −0.25, *p* < 0.001). CSF sTREM1 levels were reported as Relative Fluorescence Unit (RFU). CU, cognitively unimpaired; CI, cognitively impaired; aHV, adjusted hippocampal volume; sTREM1, soluble Triggering Receptor Expressed on Myeloid Cells 1.

To investigate the cross-sectional relationship between baseline CSF sTREM1 levels and hippocampal volumes, Pearson’s correlation tests were performed in the combined sample (including both CU and CI participants). Our analysis revealed a significant negative association between CSF sTREM1 levels and aHV (*r* = −0.3, *p* < 0.001). To ascertain whether this relationship varies by cognitive status, we conducted stratified correlation analyses in the CU and CI groups individually. Among the CU participants (*n* = 152), CSF sTREM1 levels showed a significant negative correlation with aHV (*r* = −0.32, *p* < 0.001). Similarly, in the CI group (*n* = 424), CSF sTREM1 levels were also negatively correlated with aHV (*r* = −0.25, *p* < 0.001). Figure 1 illustrates the scatter plots depicting the relationship between CSF sTREM1 levels and aHV in the CU and CI groups, visually showing the observed negative correlations.

### 3.3 CSF sTREM1 by cognitive status interaction on longitudinal hippocampal atrophy

To test the hypothesis that cognitive status influences the association between CSF sTREM1 levels and the rate of hippocampal atrophy over time, we incorporated a three-way interaction term into the linear mixed-effects model. This interaction term included continuous CSF sTREM1 levels, cognitive status (CU vs. CI), and follow-up time. The regression coefficients that indicate the associations with the longitudinal change in hippocampal volume are summarized in Table 2. Although the main effects of the predictors were indeed part of the linear mixed-effects model, we have chosen not to present them in detail in Table 2 for the sake of conciseness. This decision was made to focus the presentation on the key interactions of interest. The three-way interaction term was significant for aHV (Coefficient: −0.0006; SE: 0.0001; *p* < 0.001), indicating that the relationship between CSF sTREM1 levels and changes in hippocampal volumes over time was influenced by cognitive status.

**Table 2:** Summary of linear mixed-effect model.

Predictors	Coefficients	Standard error	*p*-values
Age × time	−0.0016	0.0003	< 0.001
Female gender × time	−0.03	0.0038	< 0.001
Education × time	0.001	0.0006	0.08
APOE4 status × time	−0.028	0.004	< 0.001
Amyloid positive × time	−0.045	0.004	< 0.001
sTREM1 × time	0.00014	0.0001	0.16
Cognitive status (CI) × time	0.1	0.027	< 0.001
sTREM1 × cognitive status (CI) × time	−0.0006	0.0001	< 0.001

Models included the main effects of predictors and their interactions with time. However, for the sake of brevity, the coefficients of the main effects were not presented. The interaction term sTREM1 × cognitive status (CI) × time was significant for aHV (coefficient = −0.0006, *p* < 0.001), suggesting that the association between baseline CSF sTREM1 levels and changes in aHV over time was influenced by cognitive status. APOE, apolipoprotein E; CI, cognitively impaired; sTREM1, soluble Triggering Receptor Expressed on Myeloid Cells 1.

**Figure 2:**
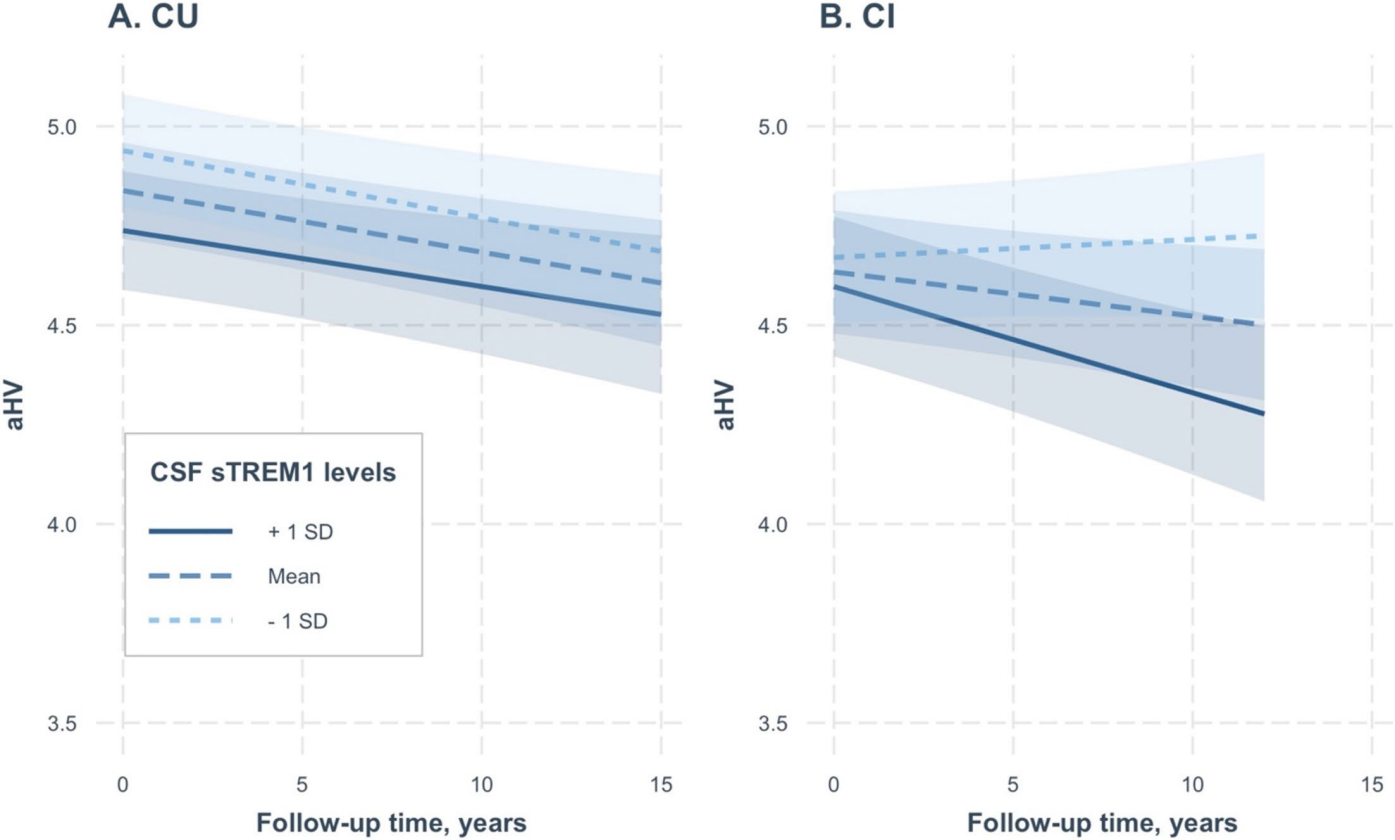
Association of continuous CSF sTREM1 levels with changes in hippocampal volumes over time in CU and CI groups. **(A,B)** Created based on two separate linear mixed-effect models, one for CU participants and the other for CI participants, respectively. CSF sTREM1 levels were treated as a continuous variable in the linear mixed-effect models, while the three levels of CSF sTREM1 (1 SD below the mean, the mean, and 1 SD above the mean) were used only for illustrative purposes. In the CU group, we did not find a significant interaction between CSF sTREM1 levels and time for aHV (coefficients: 0.00005; se: 0.0001; *p* = 0.65; Table 3). In the CI group, we found a significant interaction between CSF sTREM1 levels and time for aHV (coefficients: −0.0004; se: 0.00009; *p* < 0.0001). CU, cognitively unimpaired; CI, cognitively impaired; aHV, adjusted hippocampal volume; sTREM1, soluble Triggering Receptor Expressed on Myeloid Cells 1.

To further validate our findings and simplify the interpretation of the three-way interaction term, we conducted two linear mixed-effects models separately for the CU and CI groups. In the CU group (referenced in Table 3 and depicted in Figure 2A), we did not find a significant interaction between CSF sTREM1 levels and time for aHV (coefficients: 0.00005; se: 0.0001; *p* = 0.65). Conversely, in the CI group (as shown in Table 3; Figure 2B), we observed a significant interaction between CSF sTREM1 levels and time for aHV (coefficients: −0.0004; se: 0.00009; *p* < 0.0001).

**Table 3:** Summary of linear mixed-effect models by cognitive status.

Predictors	Coefficients	Standard error	*P*-values
CU model			
Age × time	−0.0016	0.0005	0.004
Female gender × time	−0.027	0.0055	< 0.001
Education × time	0.00055	0.0008	0.52
APOE4 status × time	−0.019	0.006	0.002
Amyloid positive × time	−0.026	0.006	< 0.001
sTREM1 × time	0.00005	0.0001	0.65
CI model			
Age × time	−0.001	0.0004	< 0.001
Female gender × time	−0.037	0.005	< 0.001
Education × time	0.0011	0.0009	0.22
APOE4 status × time	−0.03	0.005	< 0.001
Amyloid positive × time	−0.062	0.0056	< 0.001
sTREM1 × time	−0.0004	0.00009	< 0.001

Models included the main effects of predictors and their interactions with time. However, for the sake of brevity, the coefficients of the main effects were not presented. In the CU group, we did not find a significant interaction between CSF sTREM1 levels and time for aHV (coefficients: 0.00005; se: 0.0001; *p* = 0.65). Conversely, in the CI group, we observed a significant interaction between CSF sTREM1 levels and time for aHV (coefficients: −0.0004; se: 0.00009; *p* < 0.0001). CU, cognitively unimpaired; APOE, apolipoprotein E; CI, cognitively impaired; sTREM1, soluble Triggering Receptor Expressed on Myeloid Cells 1.

### 3.4 Secondary analysis

**Figure 3:**
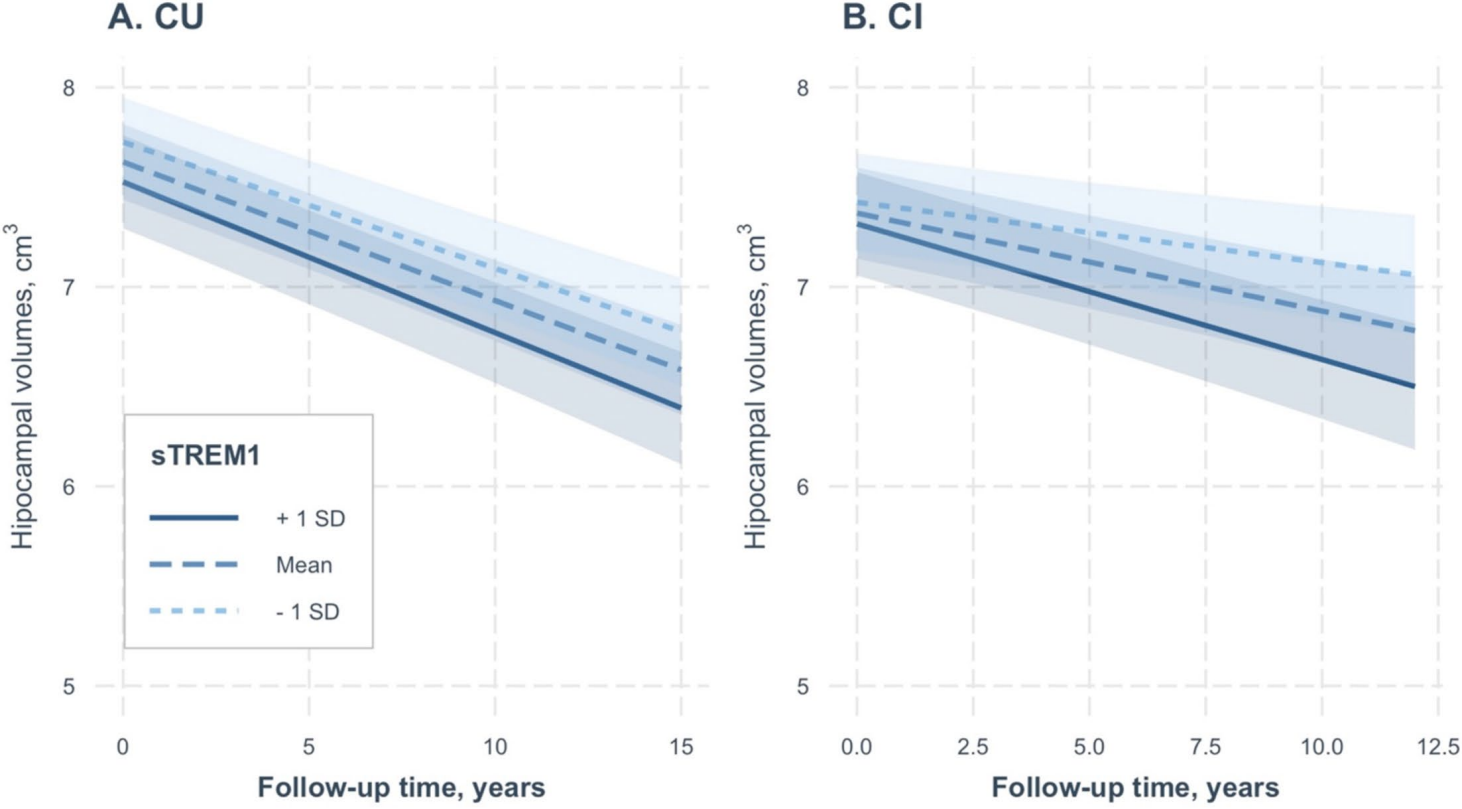
Association of continuous CSF sTREM1 levels with changes in raw hippocampal volumes over time in the CU and CI groups. CSF sTREM1 levels were treated as a continuous variable in the linear mixed-effect models, while the three levels of CSF sTREM1 (1 SD below the mean, the mean, and 1 SD above the mean) were used only for illustrative purposes. In the CU group, CSF sTREM1 levels were not associated with changes in raw hippocampal volumes over time (sTREM1 × time term: coefficients = −0.0002; se = 0.0001; *p* = 0.1; Table 4). However, we found that higher CSF sTREM1 levels were associated with a rapid reduction in raw hippocampal volumes over time in the CI group (sTREM1 × time term: coefficients = −0.0005; se = 0.0001; *p* < 0.001). CU, cognitively unimpaired; CI, cognitively impaired; sTREM1, soluble Triggering Receptor Expressed on Myeloid Cells 1.

As a sensitivity analysis, we included raw hippocampal volumes, rather than adjusted volumes, as outcomes in the linear mixed-effect models. We conducted two separate linear mixed-effect models for the CU and CI groups. The results were consistent across both groups, as depicted in Table 4 and Figures 3A,B. Higher CSF sTREM1 levels were associated with a rapid reduction in hippocampal volumes over time in the CI group (sTREM1 × time term: coefficients = −0.0005; se = 0.0001; *p* < 0.001), but not the CU group (sTREM1 × time term: coefficients = −0.0002; se = 0.0001; *p* = 0.1).

**Table 4:** Summary of linear mixed-effect models with raw hippocampal volumes as the outcome.

Predictors	Coefficients	Standard error	*P*-values
CU model			
Age × time	−0.0025	0.0006	< 0.001
Female gender × time	−0.011	0.006	0.09
Education × time	0.00036	0.001	0.72
APOE4 status × time	−0.0297	0.007	< 0.001
Amyloid positive × time	−0.0029	0.007	0.67
sTREM1 × time	−0.0002	0.0001	0.1
CI model			
Age × time	−0.001	0.00055	0.038
Female gender × time	−0.045	0.007	< 0.001
Education × time	0.001	0.001	0.32
APOE4 status × time	−0.043	0.007	< 0.001
Amyloid positive × time	−0.07	0.008	< 0.001
sTREM1 × time	−0.0005	0.0001	< 0.001

Models included the main effects of predictors and their interactions with time. However, for the sake of brevity, the coefficients of the main effects were not presented. Higher CSF sTREM1 levels were associated with a rapid reduction in raw hippocampal volumes over time in the CI group (sTREM1 × time term: coefficients = −0.0005; se = 0.0001; *p* < 0.001), but not the CU group (sTREM1 × time term: coefficients = −0.0002; se = 0.0001; *p* = 0.1). CU, cognitively unimpaired; APOE, apolipoprotein E; CI, cognitively impaired; sTREM1, soluble Triggering Receptor Expressed on Myeloid Cells 1.

We conducted correlational analyses to examine the relationship between aHV and episodic memory, as examined by Rey Auditory Verbal Learning Test (RAVLT) total score. Among the CU participants, aHV showed a significant positive correlation with RAVLT total score (*r* = 0.16, *p* = 0.04). Similarly, in the CI group aHV was also positively correlated with RAVLT total score (*r* = 0.44, *p* < 0.001). Supplementary Figure S1 illustrates the scatter plots depicting the relationship between aHV and RAVLT total score in the CU and CI groups, visually showing the observed positive correlations.

We further conducted several linear mixed-effects models examining the relationship between CSF sTREM2 levels and hippocampal atrophy for the CU and CI groups separately. We observed that higher levels of CSF sTREM2 were associated with a faster reduction in hippocampal volumes over time both in the CU (sTREM2 × time term: coefficient = −0.00002; se = 0.000006; *p* = 0.001; see Supplementary Table S1; Supplementary Figure S2A) and CI (sTREM2 × time term: coefficient = −0.00002; se = 0.000006; *p* = 0.001; Supplementary Table S1; Supplementary Figure S2B) groups.

Additionally, several linear mixed-effects models were performed to investigate the association between CSF MIF and hippocampal atrophy for the CU and CI groups separately. We found that CSF MIF levels were not associated with changes in hippocampal volumes over time either in the CU (MIF × time term: coefficient = −0.000002; se = 0.000002; *p* = 0.31; see Supplementary Table S2; Supplementary Figure S3A) and CI (MIF× time term: coefficient = −0.000003; se = 0.000003; *p* = 0.22; Supplementary Table S2; Supplementary Figure S3B) groups.

Correlational analyses were performed to examine the relationship between CSF sTREM1 levels and CSF Aβ42 levels (using continuous values) for the CU and CI groups. Among the CU participants, CSF sTREM1 levels showed a significant negative correlation with CSF Aβ42 levels (*r* = −0.2, *p* = 0.01). Similarly, in the CI group, CSF sTREM1 levels were also negatively correlated with CSF Aβ42 levels (*r* = −0.17, *p* < 0.001). Supplementary Figure S4 illustrates scatter plots depicting the relationship between CSF sTREM1 levels and CSF Aβ42 levels in the CU and CI groups, visually showing the observed negative correlations.

The comprehensive version of the results of the linear mixed-effects models for Tables 2–4, including the coefficients of the main effects and their interactions with time, is summarized in Supplementary Tables S3–S5, respectively.

## 4 Discussion

The current study had several major findings. First, in the cross-sectional analyses, baseline CSF sTREM1 levels were increased in the CI participants compared the CU participants and were negatively associated with hippocampal volumes in both CU and CI participants. Second, the linear mixed-effect model showed that the three-way interaction between CSF sTREM1, cognitive status, and follow-up time was significant for aHV, suggesting that the association between CSF sTREM1 and changes in aHV over time differed depending on the cognitive status. More specifically, our stratified analyses based on cognitive status showed that higher levels of CSF sTREM1 were associated with a more rapid rate of hippocampal atrophy in CI but not in CU participants. Our data may provide a better understanding of the interplay between neuroinflammation, brain structure changes, and cognitive decline, potentially contributing to the development of novel therapeutic strategies for AD.

Our cross-sectional analysis revealed that baseline CSF sTREM1 levels were significantly higher in CI participants compared to CU participants. This elevation in CSF sTREM1 levels in CI individuals is consistent with previous studies that have shown increased CSF and plasma sTREM1 levels in AD (Jiang et al., 2019; Del Campo et al., 2022; Hok et al., 2023), suggesting a role for neuroinflammation in the disease process (Calsolaro and Edison, 2016). We observed a negative correlation between CSF sTREM1 levels and hippocampal volumes in both CU and CI participants, suggesting that higher CSF sTREM1 levels may be associated with a greater reduction in hippocampal volume, which is a well-established marker of neurodegeneration in AD (Jack et al., 2010; Jack et al., 2018). This finding is in line with the notion that neuroinflammation contributes to neurodegeneration, such as hippocampal degeneration, observed in the AD brains (Nichols et al., 2019).

The results from our linear mixed-effect model further supported the role of sTREM1 in the progression of AD. The significant three-way interaction between CSF sTREM1, cognitive status, and follow-up time on aHV indicated that the relationship between CSF sTREM1 and longitudinal hippocampal atrophy is modified by cognitive status. This interaction underscores the importance of considering cognitive status when evaluating the impact of CSF sTREM1 on the rate of hippocampal atrophy. Our stratified analyses based on cognitive status revealed that higher levels of CSF sTREM1 were associated with a more rapid rate of hippocampal atrophy in CI participants while this association was absent in CU participants. This finding suggested that the neuroinflammatory processes, as indicated by CSF sTREM1 levels, may be more pronounced in individuals with cognitive impairment, potentially exacerbating the neurodegenerative processes. The findings from our study offered a nuanced perspective on the role of neuroinflammation in AD. The varying effects of CSF sTREM1 on hippocampal atrophy, contingent upon cognitive status, could stem from a multitude of factors, including the heightened susceptibility of neurons amidst pre-existing neurodegeneration (CI participants had decreased baseline hippocampal volumes compared CU participants), the intensification of ongoing inflammatory processes, or the disturbance of homeostatic mechanisms essential for preserving brain integrity. This may also be due to an Aβ-dependent pathway by which elevated sTREM1 accelerates neurodegeneration. For instance, a previous study showed that variants within the TREM1 gene have been linked to the accumulation of Aβ plaques in the brain, as detected through Aβ PET imaging (Jiang et al., 2016). However, caution is advised when accepting the notion that CSF sTREM1 affects neurodegeneration by an Aβ-dependent pathway. This is because a prior study failed to identify a correlation between CSF sTREM1 and CSF Aβ42 levels (Hok et al., 2023). Further studies are needed to clarify this notion. Our data suggested that targeting neuroinflammation, specifically through the modulation of sTREM1, may hold promise as a therapeutic strategy for AD (Saadipour, 2017), particularly for individuals with cognitive impairment. By reducing sTREM1 levels, it may be possible to slow the rate of hippocampal atrophy and, consequently, the progression of cognitive decline. Future studies should explore the potential of sTREM1 as a therapeutic target, including the development of interventions that can modulate sTREM1 levels in the CSF.

This study had several limitations. First, the cross-sectional and longitudinal designs of the current study limited the ability to establish causality. Future research employing experimental models or interventional studies could help clarify the causal relationships between neuroinflammation and hippocampal atrophy. Furthermore, the generalizability of our findings may be limited by the demographic characteristics of our sample. A more diverse and larger sample could provide a broader understanding of the role of CSF sTREM1 in different populations and stages of AD. In addition, exploring the underlying mechanisms by which CSF sTREM1 influences hippocampal atrophy could reveal novel targets for therapeutic intervention. Finally, serum triglycerides (TG) have been reported to be associated with systemic inflammation. Therefore, it would be of great importance to test whether TG could serve as a more accessible biomarker. Future studies are needed to address this research question.

In conclusion, our study contributes to the growing body of research highlighting the importance of neuroinflammation in the pathogenesis of AD. The association between CSF sTREM1 levels and hippocampal atrophy, particularly in CI individuals, offers a promising avenue for the development of targeted therapies and underscores the need for a nuanced approach to the management of this complex disease.

## Data Availability

Publicly available datasets were analyzed in this study. This data can be found at: The ADNI dataset (adni.loni.usc.edu).

## Footnotes

^1^ https://adni.loni.usc.edu/

^2^ https://adni.loni.usc.edu/wp-content/uploads/2024/02/ADNI_General_Procedures_Manual.pdf

^3^ https://adni.loni.usc.edu/wp-content/uploads/2024/02/ADNI_General_Procedures_Manual.pdf

^4^ www.loni.usc.edu/ADNI

## The Alzheimer’s Disease Neuroimaging Initiative

Data used in the preparation of this article were obtained from the Alzheimer’s Disease Neuroimaging Initiative (ADNI) database (adni.loni.usc.edu). As such, the investigators within the ADNI contributed to the design and implementation of ADNI and/or provided data but did not participate in the analysis or writing of this report. A complete listing of ADNI investigators can be found at: http://adni.loni.usc.edu/wp-content/uploads/how_to_apply/ADNI_Acknowledgement_List.pdf.
