# Supplementary material for: Association of CSF soluble TREM1 levels with hippocampal atrophy in cognitively impaired older adults
Source: Front Aging Neurosci. 2025 Jan 14;16:1481526. doi: 10.3389/fnagi.2024.1481526 (PMC11772463; doi:10.3389/fnagi.2024.1481526)
Supplement: Supplementary file 1 [file Data_Sheet_1.DOCX]

**Supplemental material**


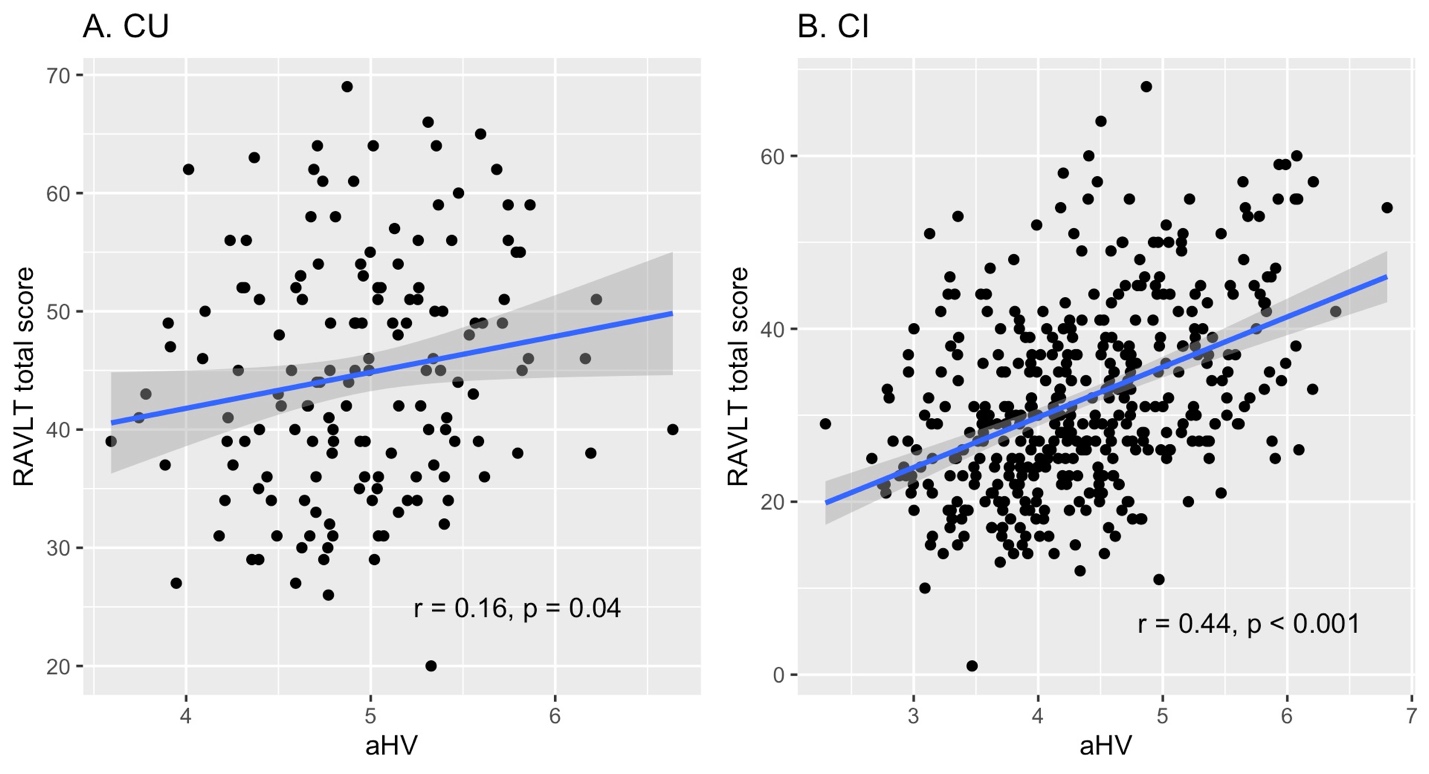


**Figure S1. Association between aHV and RAVLT total score in the CU and CI groups**.

**Table S1. Summary of linear mixed-effect model examing the relationship between CSF sTREM2 and changes in aHV over time**

| **Predictors** | **Coefficients** | **Standard error** | **P values** |
| --- | --- | --- | --- |
| **CU model** |  |  |  |
| Age × time | -0.001 | 0.0005 | 0.017 |
| Female gender × time | -0.027 | 0.005 | <0.001 |
| Education × time | 0.0009 | 0.0008 | 0.31 |
| APOE4 status × time | -0.02 | 0.006 | 0.003 |
| Amyloid positive × time | -0.03 | 0.006 | <0.001 |
| sTREM2 × time | -0.00002 | 0.000006 | 0.001 |
|  |  |  |  |
| **CI model** |  |  |  |
| Age × time | -0.002 | 0.0004 | <0.001 |
| Female gender × time | -0.03 | 0.005 | <0.001 |
| Education × time | 0.002 | 0.0009 | 0.08 |
| APOE4 status × time | -0.03 | 0.005 | <0.001 |
| Amyloid positive × time | -0.07 | 0.006 | <0.001 |
| sTREM2 × time | -0.00002 | 0.000006 | 0.001 |

Abbreviations: CU: cognitively unimpaired; APOE: apolipoprotein E; CI: cognitively impaired; sTREM2: soluble Triggering Receptor Expressed on Myeloid Cells 2.


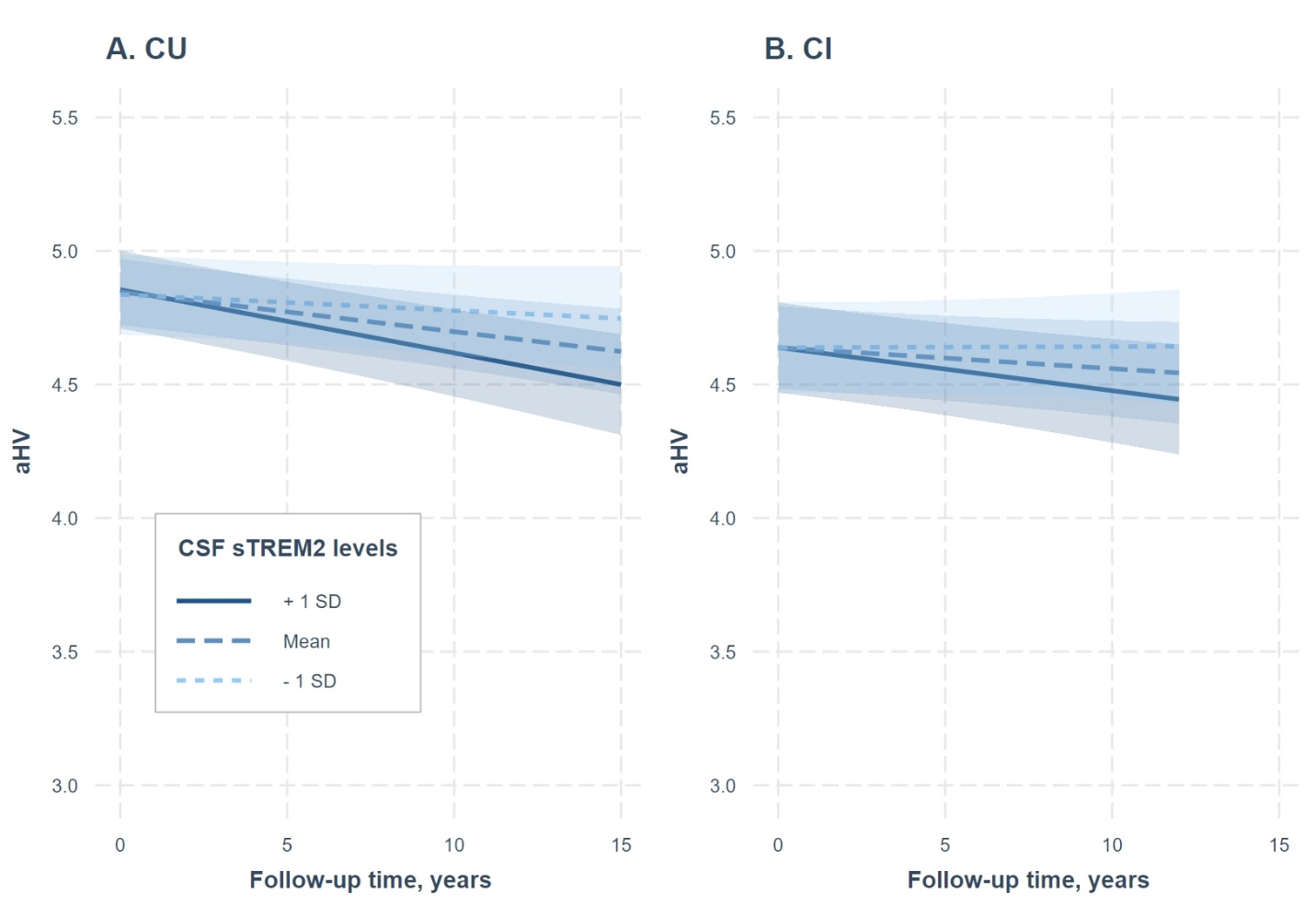


**Figure S2**. **Association of continuous CSF sTREM2 levels with changes in hippocampal volumes over time in CU and CI groups**. Figure S2.A and Figure S2.B were created based on two separate linear mixed-effect models, one for CU participants and the other for CI participants, respectively. CSF sTREM2 levels were treated as a continuous variable in the linear mixed-effect models, while the three levels of CSF sTREM2 (1 SD below the mean, the mean, and 1 SD above the mean) were used only for illustrative purposes. Abbreviations: CU: cognitively unimpaired; CI: cognitively impaired; aHV: adjusted hippocampal volume; sTREM2: soluble Triggering Receptor Expressed on Myeloid Cells 2.

**Table S2. Summary of linear mixed-effect model examing the relationship between CSF MIF and changes in aHV over time**

| **Predictors** | **Coefficients** | **Standard error** | **P values** |
| --- | --- | --- | --- |
| **CU model** |  |  |  |
| Age × time | -0.002 | 0.0005 | 0.003 |
| Female gender × time | -0.028 | 0.005 | <0.001 |
| Education × time | 0.0006 | 0.0008 | 0.46 |
| APOE4 status × time | -0.02 | 0.006 | 0.001 |
| Amyloid positive × time | -0.03 | 0.006 | <0.001 |
| MIF × time | -0.000002 | 0.000002 | 0.31 |
|  |  |  |  |
| **CI model** |  |  |  |
| Age × time | -0.002 | 0.0004 | <0.001 |
| Female gender × time | -0.03 | 0.005 | <0.001 |
| Education × time | 0.0016 | 0.0009 | 0.09 |
| APOE4 status × time | -0.03 | 0.005 | <0.001 |
| Amyloid positive × time | -0.06 | 0.006 | <0.001 |
| MIF × time | -0.000003 | 0.000003 | 0.22 |

Abbreviations: CU: cognitively unimpaired; APOE: apolipoprotein E; CI: cognitively impaired; MIF: macrophage migration inhibitory factor.


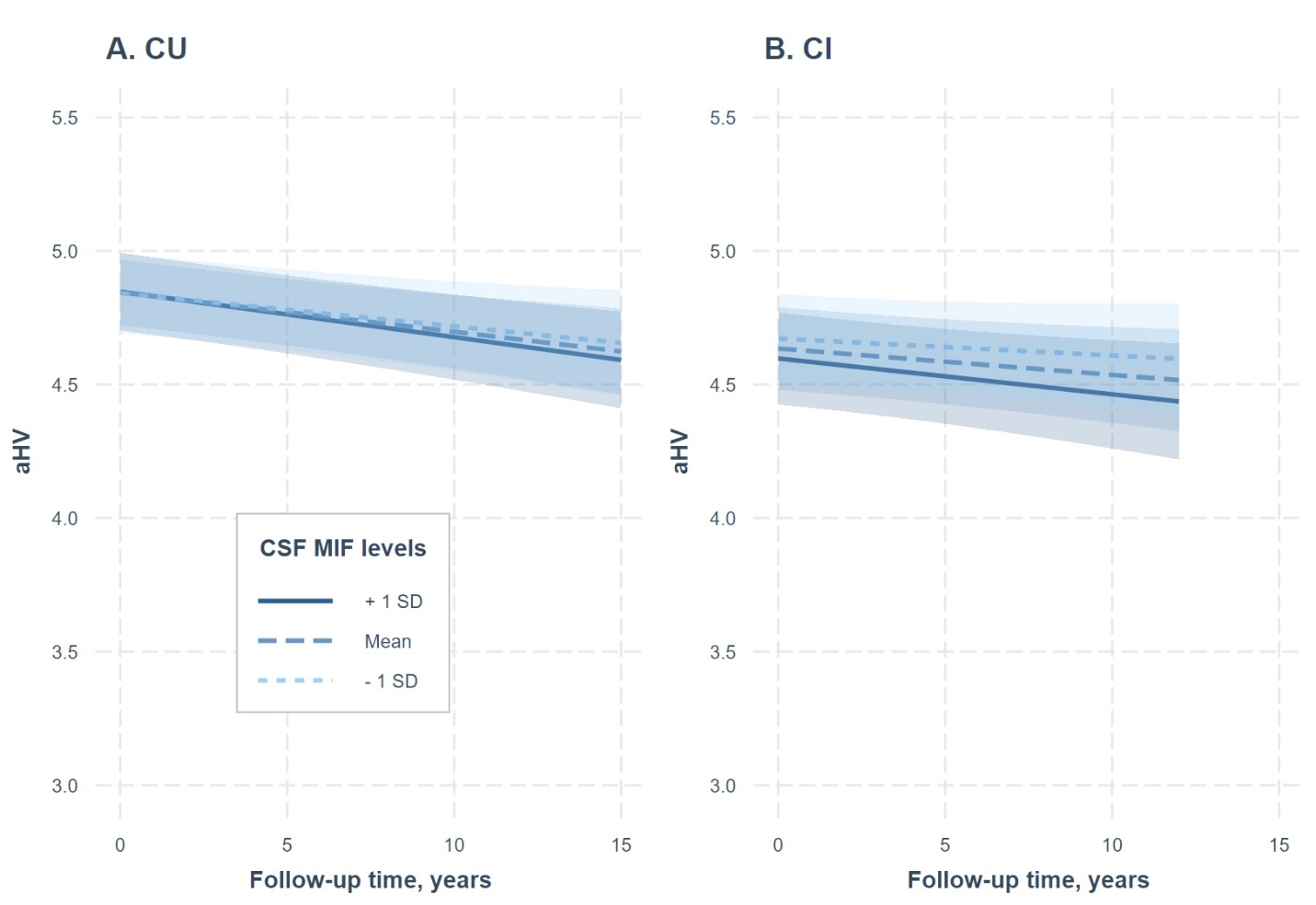


**Figure S3**. **Association of continuous CSF MIF levels with changes in hippocampal volumes over time in CU and CI groups**. Figure S3.A and Figure S3.B were created based on two separate linear mixed-effect models, one for CU participants and the other for CI participants, respectively. CSF MIF levels were treated as a continuous variable in the linear mixed-effect models, while the three levels of CSF MIF (1 SD below the mean, the mean, and 1 SD above the mean) were used only for illustrative purposes. Abbreviations: CU: cognitively unimpaired; CI: cognitively impaired; aHV: adjusted hippocampal volume; MIF: macrophage migration inhibitory factor.


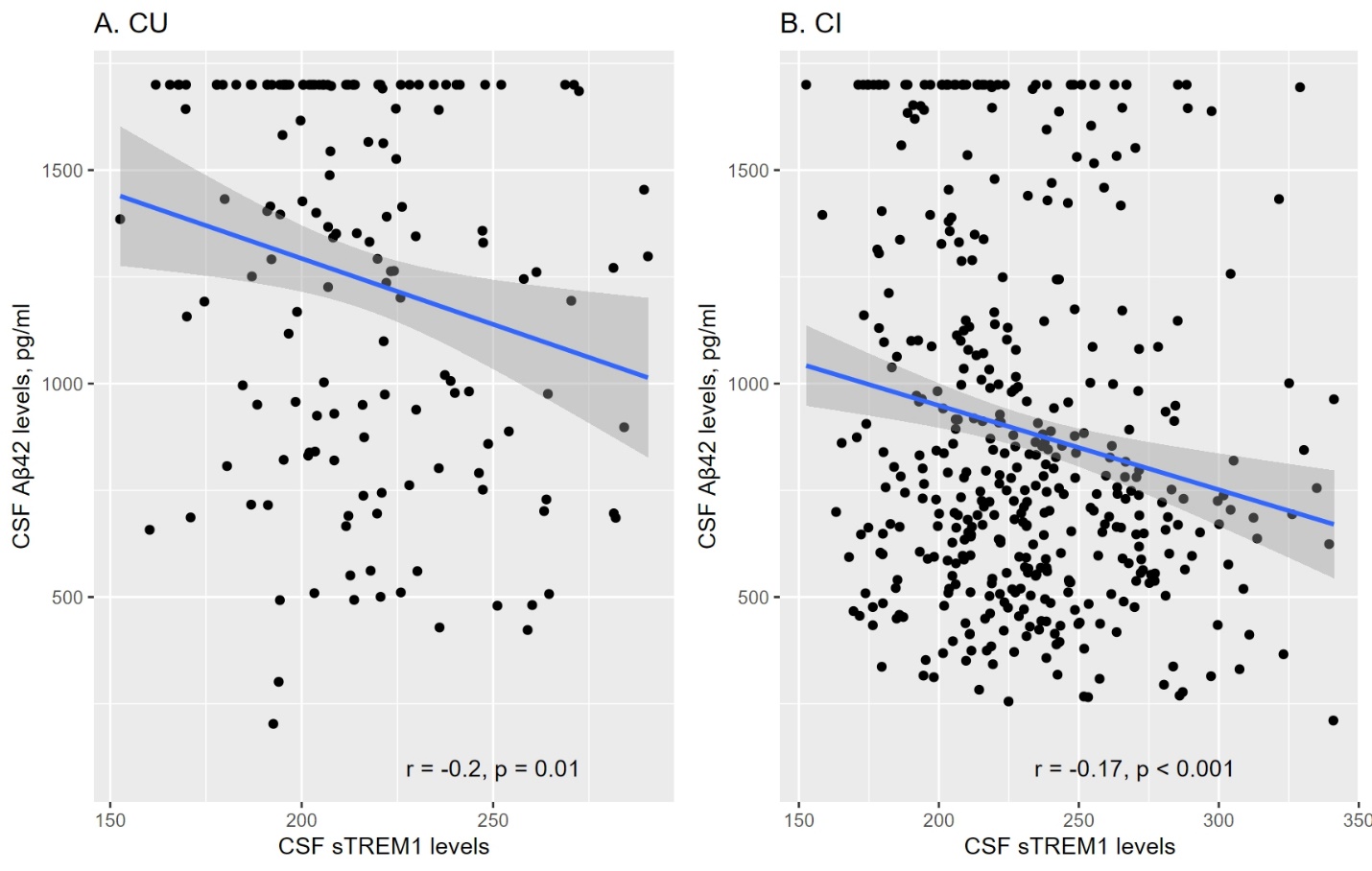


**Figure S4. Association between CSF sTREM1 levels and CSF Aβ42 levels in the CU and CI groups**.

**Table S3. Summary of linear mixed-effect model (comprehensive version of Table 2)**

| Predictors | Coefficients | Standard error | P values |
| --- | --- | --- | --- |
| Age | -0.04 | 0.004 | <0.001 |
| Time | 0.06 | 0.03 | 0.05 |
| Female gender | 0.17 | 0.058 | 0.003 |
| Education | -0.02 | 0.01 | 0.03 |
| APOE4 carriers | -0.197 | 0.06 | 0.002 |
| Amyloid positive | -0.2 | 0.07 | 0.001 |
| sTREM1 | -0.002 | 0.002 | 0.3 |
| Cognitive status (CI) | -0.63 | 0.45 | 0.16 |
| sTREM1 × Cognitive status (CI) | 0.0005 | 0.002 | 0.79 |
| Age × time | -0.0016 | 0.0003 | < 0.001 |
| Female gender × time | -0.03 | 0.0038 | < 0.001 |
| Education × time | 0.001 | 0.0006 | 0.08 |
| APOE4 carriers × time | -0.028 | 0.004 | < 0.001 |
| Amyloid positive × time | -0.045 | 0.004 | < 0.001 |
| sTREM1 × time | 0.00014 | 0.0001 | 0.16 |
| Cognitive status (CI) × time | 0.1 | 0.027 | < 0.001 |
| sTREM1 × cognitive status (CI) × time | -0.0006 | 0.0001 | < 0.001 |

Abbreviations: APOE: apolipoprotein E; CI: cognitively impaired; sTREM1: soluble Triggering Receptor Expressed on Myeloid Cells 1.

**Table S4. Summary of linear mixed-effect models by cognitive status (comprehensive version of Table 3)**

| **Predictors** | **Coefficients** | **Standard error** | **P values** |
| --- | --- | --- | --- |
| **CU model** |  |  |  |
| Age | -0.03 | 0.007 | <0.001 |
| Time | 0.08 | 0.04 | 0.06 |
| Female gender | 0.2 | 0.08 | 0.016 |
| Education | -0.03 | 0.015 | 0.02 |
| APOE4 carriers | -0.14 | 0.096 | 0.15 |
| Amyloid positive | 0.04 | 0.08 | 0.63 |
| sTREM1 | -0.003 | 0.001 | 0.02 |
| Age × time | -0.0016 | 0.0005 | 0.004 |
| Female gender × time | -0.027 | 0.0055 | < 0.001 |
| Education × time | 0.00055 | 0.0008 | 0.52 |
| APOE4 carriers × time | -0.019 | 0.006 | 0.002 |
| Amyloid positive × time | -0.026 | 0.006 | < 0.001 |
| sTREM1 × time | 0.00005 | 0.0001 | 0.65 |
|  |  |  |  |
| **CI model** |  |  |  |
| Age | -0.045 | 0.005 | <0.001 |
| Time | 0.17 | 0.03 | <0.001 |
| Female gender | 0.15 | 0.07 | 0.04 |
| Education | -0.02 | 0.01 | 0.12 |
| APOE4 carriers | -0.2 | 0.075 | 0.008 |
| Amyloid positive | -0.32 | 0.08 | <0.001 |
| sTREM1 | -0.001 | 0.001 | 0.32 |
| Age × time | -0.001 | 0.0004 | < 0.001 |
| Female gender × time | -0.037 | 0.005 | < 0.001 |
| Education × time | 0.0011 | 0.0009 | 0.22 |
| APOE4 carriers × time | -0.03 | 0.005 | < 0.001 |
| Amyloid positive × time | -0.062 | 0.0056 | < 0.001 |
| sTREM1 × time | -0.0004 | 0.00009 | < 0.001 |

Abbreviations: CU: cognitively unimpaired; APOE: apolipoprotein E; CI: cognitively impaired; sTREM1: soluble Triggering Receptor Expressed on Myeloid Cells 1.

**Table S5. Summary of linear mixed-effect models with raw hippocampal volumes as the outcome (comprehensive version of Table 4)**

| **Predictors** | **Coefficients** | **Standard error** | **P values** |
| --- | --- | --- | --- |
| **CU model** |  |  |  |
| Age | -0.04 | 0.01 | <0.001 |
| Time | 0.16 | 0.05 | 0.003 |
| Female gender | -0.49 | 0.13 | <0.001 |
| Education | 0.006 | 0.02 | 0.81 |
| APOE4 carriers | -0.07 | 0.15 | 0.6 |
| Amyloid positive | 0.02 | 0.1 | 0.86 |
| sTREM1 | -0.003 | 0.002 | 0.13 |
| Age × time | -0.0025 | 0.0006 | < 0.001 |
| Female gender × time | -0.011 | 0.006 | 0.09 |
| Education × time | 0.00036 | 0.001 | 0.72 |
| APOE4 carriers × time | -0.0297 | 0.007 | < 0.001 |
| Amyloid positive × time | -0.0029 | 0.007 | 0.67 |
| sTREM1 × time | -0.0002 | 0.0001 | 0.1 |
|  |  |  |  |
| **CI model** |  |  |  |
| Age | -0.07 | 0.007 | <0.001 |
| Time | 0.13 | 0.047 | 0.005 |
| Female gender | -0.56 | 0.1 | <0.001 |
| Education | 0.0004 | 0.018 | 0.98 |
| APOE4 carriers | -0.3 | 0.1 | 0.004 |
| Amyloid positive | -0.4 | 0.1 | <0.001 |
| sTREM1 | -0.001 | 0.001 | 0.32 |
| Age × time | -0.001 | 0.00055 | 0.038 |
| Female gender × time | -0.045 | 0.007 | < 0.001 |
| Education × time | 0.001 | 0.001 | 0.32 |
| APOE4 carriers × time | -0.043 | 0.007 | < 0.001 |
| Amyloid positive × time | -0.07 | 0.008 | < 0.001 |
| sTREM1 × time | -0.0005 | 0.0001 | < 0.001 |

Abbreviations: CU: cognitively unimpaired; APOE: apolipoprotein E; CI: cognitively impaired; sTREM1: soluble Triggering Receptor Expressed on Myeloid Cells 1.
